# Antisense suppression of the nonsense mediated decay factor *Upf3b* as a potential treatment for diseases caused by nonsense mutations

**DOI:** 10.1186/s13059-017-1386-9

**Published:** 2018-01-15

**Authors:** Lulu Huang, Audrey Low, Sagar S. Damle, Melissa M. Keenan, Steven Kuntz, Susan F. Murray, Brett P. Monia, Shuling Guo

**Affiliations:** Ionis Pharmaceuticals, Inc., Carlsbad, California USA

**Keywords:** NMD, PTC, ASO, Upf3b, RNA, Hemophilia

## Abstract

**Background:**

About 11% of all human genetic diseases are caused by nonsense mutations that generate premature translation termination codons (PTCs) in messenger RNAs (mRNA). PTCs not only lead to the production of truncated proteins, but also often result in  decreased mRNA abundance due to  nonsense-mediated mRNA decay (NMD). Although pharmacological inhibition of NMD could be an attractive therapeutic approach for the treatment of diseases caused by nonsense mutations, NMD also regulates the expression of 10–20% of the normal transcriptome.

**Results:**

Here, we investigate whether NMD can be inhibited to stabilize mutant mRNAs, which may subsequently produce functional proteins, without having a major impact on the normal transcriptome. We develop antisense oligonucleotides (ASOs) to systematically deplete each component in the NMD pathway. We find that ASO-mediated depletion of each NMD factor elicits different magnitudes of NMD inhibition in vitro and are differentially tolerated in normal mice. Among all of the NMD factors, *Upf3b* depletion is well tolerated, consistent with previous reports that UPF3B is not essential for development and regulates only a subset of the endogenous NMD substrates. While minimally impacting the normal transcriptome, *Upf3b*-ASO treatment significantly stabilizes the PTC-containing dystrophin mRNA in mdx mice and coagulation factor IX mRNA in a hemophilia mouse model.

Furthermore, when combined with reagents promoting translational read-through, *Upf3b*-ASO treatment leads to the production of functional factor IX protein in hemophilia mice.

**Conclusions:**

These data demonstrate that ASO-mediated reduction of the NMD factor *Upf3b* could be an effective and safe approach for the treatment of diseases caused by nonsense mutations.

**Electronic supplementary material:**

The online version of this article (doi:10.1186/s13059-017-1386-9) contains supplementary material, which is available to authorized users.

## Background

About 11% of all human genetic diseases are caused by nonsense mutations that generate premature translation termination codons (PTCs) in messenger RNAs (mRNA) [1, 2]. PTCs often inactivate gene function due to the production of truncated proteins and usually lead to a significant decrease in mRNA abundance due to degradation by the nonsense-mediated mRNA decay (NMD) pathway [3, 4]. In human diseases, the role of NMD can be twofold: on one hand, if the PTC results in production of a dominant-negative, truncated protein, NMD can protect cells by eliminating the aberrant transcripts; on the other hand, if the truncated protein is partially functional, NMD can lead to a more severe disease [5–7].

The development of therapeutic approaches for diseases caused by nonsense mutations has focused on small-molecule read-through agents [8, 9]. The goal of this type of therapy is to induce the translation machinery to recode a PTC into a sense codon so that translation continues in the correct reading frame to complete the synthesis of a full-length, potentially functional protein. As of today, no read-through therapy has received final approval for the treatment of diseases caused by nonsense mutations due to the lack of sufficient efficacy and/or safety. This is in part because of the low abundance of mRNA substrates available for translational read-through due to their degradation by NMD [8–11]. It has been shown that NMD attenuation by small molecule inhibitors or small interfering RNAs (siRNA) targeting NMD factors significantly enhances the efficacy of translational read-through drugs in cell lines derived from cystic fibrosis patients carrying a nonsense mutation in the *CFTR* gene [11], as well as in a mouse model of the lysosomal storage disease mucopolysaccharidosis I-Hurler (MPS I-H) caused by a PTC in the *Idua* gene locus [12]. Inhibition of NMD alone also partially restores protein function by stabilizing PTC-containing mRNAs when the truncated proteins are functional as shown in Ullrich disease patient-derived fibroblasts [13, 14] and in a mouse model for neuronal ceroid lipofuscinosis [15, 16].

More than twenty proteins have been reported to play a role in NMD [4, 17–19]. The recognition and degradation of mRNAs with PTCs is mediated by sequential remodeling of protein–RNA complexes [17–19]. In mammals, the current model suggests that a PTC is recognized when the stop codon is distant from the poly(A) tail so that the translation termination factor ERF3 is recruited to the ribosome at a PTC, but binds UPF1 instead of PABP as during normal translation termination [18, 20]. This forms the SMG1–UPF1–eRF1–eRF3 (SURF) complex that then interacts with UPF2 and/or UPF3B, which, in some cases, is facilitated by the exon junction complex (EJC), to trigger UPF1 activation by phosphorylation [18, 20]. The phosphorylation of UPF1 is mediated by the kinase SMG1, which is regulated by SMG8 and SMG9 [18, 20]. Once UPF1 is activated, the mRNA is tagged for degradation. Phosphorylated UPF1 then recruits SMG6, which cleaves the mRNA near the PTC. The 3΄ RNA fragment is then rapidly degraded by XRN1 and the 5΄ fragment may be digested by the exosome [18, 20]. In addition, UPF1 also recruits the SMG5–SMG7 heterodimer that in turn recruits the CCR4-NOT complex to induce mRNA deadenylation-dependent decapping and subsequent XRN1-mediated degradation [18, 20].

Beyond its role in RNA surveillance, NMD is a post-transcriptional regulatory pathway that regulates 10–20% of the normal transcriptome across many species [4, 17–19]. Therefore, inhibition of the NMD pathway could have catastrophic effects on an organism, which is supported by the fact that several NMD factors are essential for early embryonic development in mouse [21–25]. Several lines of evidence suggest that NMD is not a single biochemical pathway in higher eukaryotes, but rather a pathway with several branches [18]. Three branches of the NMD pathway diverging at the stage of PTC recognition were reported—UPF2-independent, EJC-independent, and UPF3B-independent branches—each of which only regulates a subset of the endogenous NMD substrates [26–28]. At the step of RNA destruction, several studies show that NMD substrate RNAs can be degraded through either SMG6-mediated endonucleolytic degradation or SMG5-SMG7-mediated degradation [29–32]. These branch-specific NMD factors could be potential therapeutic targets for diseases caused by nonsense mutations. However, it remains unclear if NMD can be effectively inhibited to stabilize disease-causing PTC transcripts with minimum impact on the normal transcriptome, resulting in an acceptable therapeutic index.

Here, we sought to identify those NMD components that could be depleted to effectively inhibit NMD to alleviate the phenotype of PTC-related genetic diseases, while simultaneously causing minimum toxicity to the organism. We used antisense oligonucleotides (ASOs) as tools to address this question. ASOs bind specifically to their RNA targets through Watson-Crick base pairing to form DNA–RNA heteroduplexes. These DNA–RNA heteroduplexes are substrates for the ubiquitous endonuclease RNase H1, which mediates the degradation of the target RNA strand [33, 34]. ASOs have proven to be specific, potent, and well tolerated treatment approaches for cardiovascular, metabolic, neurological, and severe genetic diseases and cancer [35].

In this study, we developed ASOs to specifically deplete mRNAs encoding mouse core NMD factors, the UPF and SMG proteins, to evaluate the efficacy and safety of NMD inhibition. Among the ten NMD factors targeted, we found that the ASO-mediated depletion of *Upf3b* efficiently suppressed NMD on certain disease-causing mRNAs and had a minimal global impact on the transcriptome. Our results suggest that targeting *UPF3B* with ASOs might be a viable approach for inhibiting NMD to ameliorate human diseases caused by nonsense mutations.

## Results

### Identification of three categories of NMD regulators using ASOs

**Fig. 1:**
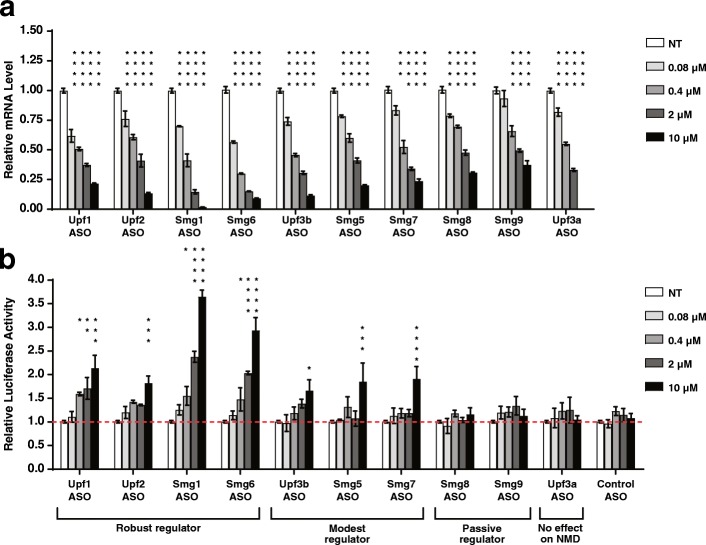
Nonsense-mediated degradation of a β-*GLOBIN* luciferase reporter is inhibited by ASOs targeting NMD factors. Mouse MHT cells stably expressing a PTC-containing β-*GLOBIN* Renilla luciferase reporter were treated with ASOs targeting mouse NMD factors *Upf1*, *Upf2*, *Smg1*, *Smg6*, *Upf3b*, *Smg5*, *Smg7*, *Smg8*, *Smg9*, or *Upf3a*, by free uptake at the indicated concentrations for 72 h. Results are presented as means ± standard errors (n = 3). **a** qPCR analysis of the mRNA levels of each NMD factor after ASO treatment. Mouse *Gapdh* mRNA was used as endogenous control. The mRNA level of each NMD factor in untreated (NT) MHT cells was set as 1. **b** Relative luciferase activity after ASO treatment. The luciferase activity from the PTC-containing β-*GLOBIN* Renilla construct was normalized to the Firefly luciferase signal, which was also stably expressed in the MHT cells. Luciferase activity in untreated MHT cells was set as 1. Results were grouped in three categories: Robust, Modest or Passive regulators. Statistical significance was determined using a two-way ANOVA and Dunnett’s multiple comparison test in Prism. All groups were compared to NT group within each measurement. * *p* < 0.05; ** *p* < 0.01; *** *p* < 0.001; **** *p* < 0.0001

Active and well tolerated ASOs were identified against ten mouse NMD factors: *Upf1*, *Upf2*, *Upf3a*, *Upf3b*, *Smg1*, *Smg5*, *Smg6*, *Smg7*, *Smg8*, and *Smg9* by screening multiple ASOs targeting each mRNA (data not shown). To evaluate the effect on NMD pathway activity by ASOs targeting individual NMD factors, we stably expressed either the wild-type (WT) or PTC-containing β*-GLOBIN* luciferase reporter, a well characterized NMD reporter system [36], in the mouse liver MHT cell line. These MHT cells were then treated with ASOs targeting each of the NMD factors via ASO free uptake. Careful dose-response experiments were conducted to determine the efficiency of ASO-mediated target knockdown and the impact of factor depletion on NMD activity. mRNA abundance of each target was quantified by isolation of total RNA followed by quantitative polymerase chain reaction (qPCR) analysis (Fig. 1a). Western blot analysis demonstrated that, generally, ASO-mediated mRNA reduction was correlated with reduction at the protein level (Fig. 1a and Additional file 1: Figure S1). NMD inhibition was evaluated using the reporter luciferase assay and by qPCR analysis of endogenous NMD substrates [37, 38].

**Fig. 2:**
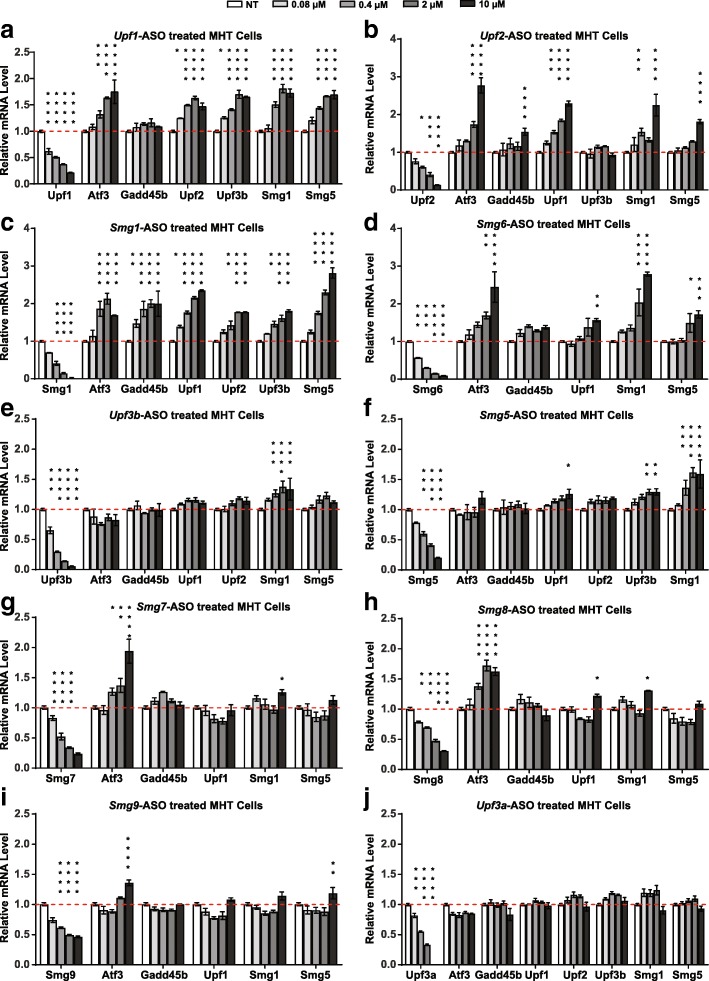
Nonsense-mediated degradation of endogenous NMD substrates is inhibited by ASOs targeting NMD factors. Mouse MHT cells were treated with ASOs targeting mouse NMD factors by free uptake at the indicated concentrations. mRNA levels of the ASO-targeted NMD factors and  endogenous NMD substrates *Atf3*, *Gadd45b*, *Upf1*, *Upf2,Upf3b*, *Smg1*, and *Smg5* were measured by qPCR analysis. Results are presented as means ± standard errors (n = 3). Mouse *Gapdh* mRNA was used as endogenous control. The mRNA level of each gene in untreated (NT) MHT cells was set as 1. **a** *Upf1*-ASO treated cells. **b** *Upf2*-ASO treated cells. **c** *Smg1*-ASO treated cells. **d** *Smg6*-ASO treated cells. **e** *Upf3b*-ASO treated cells. **f** *Smg5*-ASO treated cells. **g** *Smg7*-ASO treated cells. **h** *Smg8*-ASO treated cells. **i** *Smg9*-ASO treated cells. **j** *Upf3a*-ASO treated cells. Statistical significance was determined using a two-way ANOVA and Dunnett’s multiple comparison test in Prism. All groups were compared to NT group within each measurement. * *p* < 0.05; ** *p* < 0.01; *** *p* < 0.001; **** *p* < 0.0001

We found that the depletion of different NMD factors had different impacts on NMD activity in MHT cells. We classified these ten NMD factors into three categories based on the amount of NMD pathway inhibition that result from their depletion. The first category contains the “robust” NMD regulators *Upf1*, *Upf2*, *Smg1*, and *Smg6*. ASO-mediated depletion of these NMD factors by > 50% significantly inhibited NMD as shown by two- to fourfold increases in luciferase signal from the PTC-containing reporter (Fig. 1 and Additional file 1: Figure S1). In addition, these NMD factors regulated the expression of most of the endogenous NMD substrates tested (Fig. 2a–d), reflecting their central roles in the NMD pathway. This effect was specific to the PTC-containing mRNAs, as there was no effect on luciferase activity from the WT β*-GLOBIN* luciferase reporter upon treatment of cells with ASOs targeting these four factors (Additional file 1: Figure S2). A negative control ASO was included in all experiments and did not show any significant effect on either the levels of the NMD factors or the NMD substrates (Additional file 1: Figure S3).

The second category contains the “modest” NMD regulators *Upf3b*, *Smg5*, and *Smg7*. Only when these NMD factors were depleted by > 70% was there an increase in signal from the PTC-containing reporter (Fig. 1 and Additional file 1: Figure S1). These factors also regulated fewer endogenous NMD substrates and to a lesser extent than the robust regulators (Fig. 2e–g). We categorized *Smg8* and *Smg9* as “passive” NMD regulators, as their depletion did not significantly alter the luciferase signal from the PTC-containing reporter (Fig. 1 and Additional file 1: Figure S1) and only slightly affected the levels of a few endogenous NMD substrates tested (Fig. 2h and i). Finally, depletion of *Upf3a* by > 99% did not inhibit reporter NMD and did not alter the abundance of the endogenous NMD substrates tested (Figs. 1 and 2j). While these results are consistent with a recent report that UPF3A acts primarily as an NMD inhibitor [25], we did not observe NMD activation with ASO-mediated *Upf3a* depletion in mouse MHT cells (Figs. 1 and 2j).

### ASO-mediated *Upf3b* depletion is well tolerated in normal mice

Several NMD factors are essential for mammalian development. Complete knockout of *Upf1*, *Upf2*, *Upf3a*, *Smg1*, and *Smg6* in mouse causes early embryonic lethality [21–25]. Heterozygote animals are normal, however, with fully functional NMD. Depletion of these NMD factors in adult animals has not been fully investigated. To address the question of whether there is a tolerable level of NMD inhibition in adult animals that is sufficient to alleviate disease phenotypes caused by nonsense mutations, we first conducted dose-response experiments in normal mice with ASOs targeting mRNAs encoding each of the ten NMD factors. Animals were dosed subcutaneously with ASOs twice a week for four weeks. Body weights were monitored throughout the study. At four weeks, necropsy was conducted and organ weights, as well as blood chemistry analyses including alanine aminotransferase (ALT), aspartate aminotransferase (AST), total bilirubin (T. Bil), albumin (Alb), and blood urea nitrogen (BUN) levels, parameters indicative of liver and kidney function, were measured (Additional file 2: Table S1) [39]. The efficacies of NMD factor depletions in mouse livers were evaluated at both the RNA and protein levels (Additional file 2: Table S1 and data not shown).

As expected, ASO-mediated depletion of the robust NMD regulators was less well tolerated compared to the depletion of the modest and passive NMD regulators. Tolerability somewhat correlated with the degree of NMD pathway inhibition upon their depletion, as measured by upregulation of endogenous NMD substrates (Additional file 2: Table S1 and data not shown). Among all the NMD factors, the depletion of the robust NMD regulators *Upf2* and *Smg1* were least tolerated. Reduction of *Upf2* mRNA by 80–90% caused mild to severe toxicity, demonstrated by less body weight gain and elevated plasma ALT and AST levels compared to control-ASO treated mice (Additional file 2: Table S1). This is consistent with the observation that *Upf2* liver-specific deletion leads to liver injury and steatosis [40]. Up to 60% depletion of *Upf2* mRNA in mouse liver was tolerated (Additional file 2: Table S1). For *Smg1*, 60–70% mRNA depletion induced elevation of plasma ALT and AST levels (Additional file 2: Table S1). For *Upf1*, the target reduction in the liver plateaued at approximately 50%, as animals dosed weekly at 12.5, 25, or 50 mg/kg/week all had *Upf1* mRNA and protein levels reduced to similar extents of about 50% of control levels (Additional file 2: Table S1 and data not shown). Although multiple additional ASOs were evaluated, none reduced *Upf1* levels by > 50% in mouse liver (data not shown). Depletion of the final robust NMD factor, *Smg6*, was relatively well tolerated in normal mice compared to the depletion of the other robust NMD regulators, as > 90% mRNA reduction only resulted in slightly increased liver weights (by 30% compared to control animals) and ALT/AST levels (by approximately threefold compared to control animals) (Additional file 2: Table S1). This could be because SMG6 only functions in one of the two potentially redundant RNA degradation pathways that destruct mRNAs targeted for NMD [30].

**Fig. 3:**
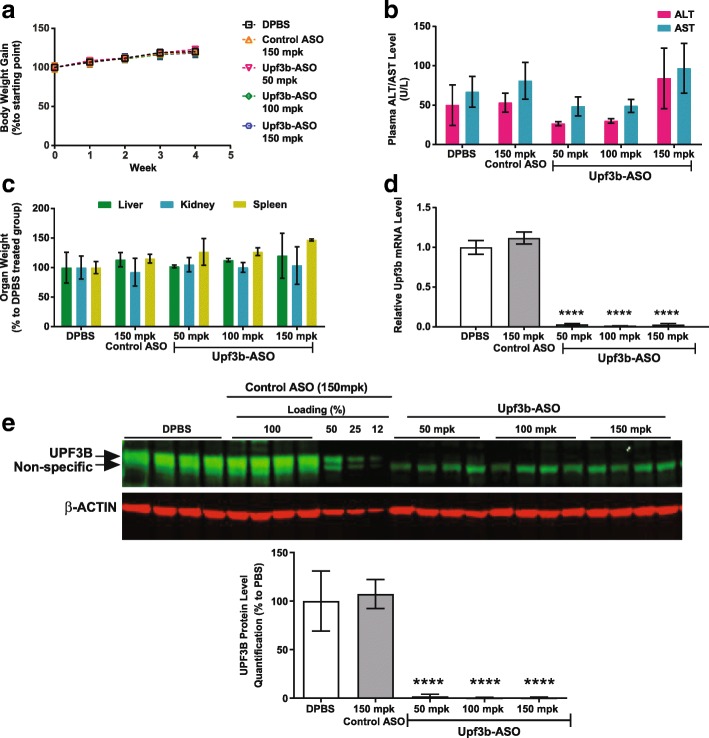
ASO-mediated *Upf3b* depletion is well tolerated in normal mice. Mice (n = 4) were treated with an *Upf3b*-ASO at 50, 100, or 150 mg/kg/week. DPBS and a scrambled ASO dosed at 150 mg/kg/week were used as controls. Animals were dosed twice a week for total of eight doses in a 4-week period. Necropsy was performed 48 h after the last dose of ASO. Results are presented as means ± standard errors. **a** Body weights of the mice over the course of the study. **b** Plasma ALT and AST levels as measured by clinical analyzer at necropsy. **c** Liver, kidney, and spleen weights measured at necropsy. **d** qPCR analysis of *Upf3b* mRNA levels in mouse liver samples. Mouse *Gapdh* mRNA was used as endogenous control. *Upf3b* mRNA level in DPBS treated animals was set as 1. **e** Western blot analysis of UPF3B protein levels in mouse liver samples with UPF3B-specific antibody. *Top*: Odyssey (LI-COR) images of the western blot. *Bottom*: Image studio quantification of the western blot image in the upper panel. β-ACTIN protein levels were used as loading controls. UPF3B protein level in DPBS treated animals was set as 100%. Statistical significance was determined using a two-way ANOVA and Dunnett’s multiple comparison test in Prism. All groups were compared to DPBS-treated mice. **** *p* < 0.0001. mpk mg/kg

Depletion of the modest and passive NMD regulators by 80–90% was generally well tolerated (Additional file 2: Table S1). Interestingly, our *Upf3b*-targeting ASO showed high efficacy for target depletion at the RNA and protein level and had one of the best tolerability profiles (Fig. 3 and Additional file 2: Table S1). In all *Upf3b*-ASO treated mice, *Upf3b* mRNA and protein levels were depleted by > 95% compared to control animals and the animals had normal body weight gain, organ weights, and blood chemistry readings (Fig. 3 and Additional file 2: Table S1). These findings are consistent with the previous report that *Upf3b*-null mice are viable and fertile [38, 59].

Overall, we found that the depletion of the robust NMD factors was less well tolerated than the depletion of the modest or passive NMD factors. We hypothesized that the depletion of the robust NMD factors would elicit strong inhibition of NMD and significantly stabilize disease-associated PTC-containing mRNAs, but would likely lead to significant changes in the normal transcriptome and therefore would not be well tolerated as a therapeutic approach. In contrast, the depletion of the modest NMD factors might be sufficient to stabilize the disease-associated PTC mRNAs while having less impact on the normal transcriptome and therefore could be potential therapeutic targets for diseases caused by nonsense mutations. To test this hypothesis, we evaluated ASOs targeting mRNAs encoding NMD factors in mouse disease models.

### *Upf3b*-ASO treatment stabilizes dystrophin PTC-containing mRNA

**Fig. 4:**
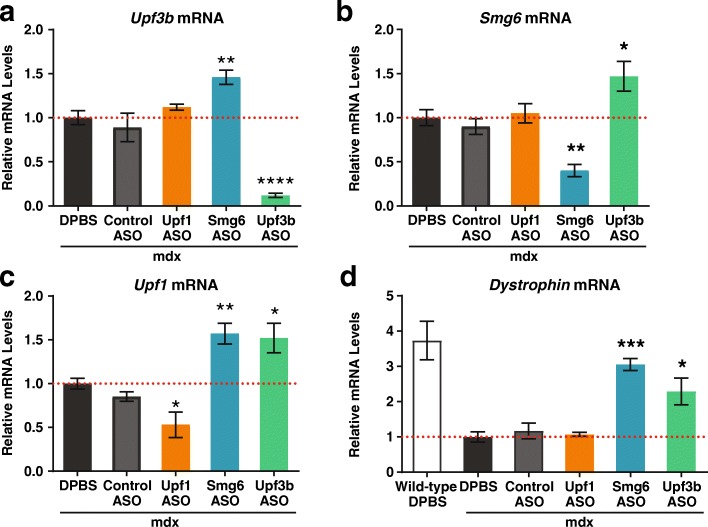
Dystrophin PTC-containing mRNA is stabilized in mdx mice treated with *Upf3b*- and *Smg6*-ASOs. Male mdx mice (aged five weeks; *n* = 4) were treated with DPBS, Control ASO (100 mg/kg/week), *Upf1*-ASO (50 mg/kg/week), *Smg6*-ASO (100 mg/kg/week), or *Upf3b*-ASO (100 mg/kg/week) for five weeks. Animals were sacrificed 48 h after the last ASO dose. Total RNA was isolated from mouse TA muscle. mRNA levels of NMD factors and dystrophin were analyzed by qPCR and normalized to total RNA as measured using Ribogreen. The expression levels in DPBS-treated mouse TA muscle were set as 1. Results are presented as means ± standard errors. **a** *Upf3b* mRNA levels. **b** *Smg6* mRNA levels. **c** *Upf1* mRNA levels. **d** Dystrophin mRNA levels. Dystrophin mRNA level in wild-type DPBS treated mice was included as control. Statistical significance was determined using a one-way ANOVA and Dunnett’s multiple comparison test in Prism. All groups were compared to DPBS-treated mdx mouse group. * *p* < 0.05; ** *p* < 0.01; *** *p* < 0.001; **** *p* < 0.0001

Since the *Upf3b*-ASO effectively depleted cells of *Upf3b* and was well tolerated in normal animals, we tested the *Upf3b*-ASO in mdx mice, a model of Duchenne muscular dystrophy (DMD). DMD is an X-linked recessive disorder that affects 1 in 3500 live-born males and is caused by mutations in the dystrophin gene [41]. Patients are characterized by a lack of dystrophin protein in the sarcolemma [42]. Nonsense mutations in the dystrophin gene are observed in approximately 15% of dystrophinopathy patients [43]. The mdx mice carry a nonsense mutation (CAA to TAA) in exon 23 of the dystrophin gene, which leads to loss of dystrophin expression; the phenotype of these mice resembles DMD disease phenotypes [44]. We treated five-week-old mdx mice with an ASO targeting *Upf3b* twice a week for five weeks. As positive controls for inhibiting NMD, we also tested both an *Upf1*- and a *Smg6*-ASO. UPF1 plays a central role in the NMD pathway [18]. However, we could not achieve > 50% *Upf1* mRNA knockdown with ASO treatment in normal mouse muscle tissue (data not shown). Therefore, we also included the *Smg6*-ASO, which depleted *Smg6* mRNA by at least 70% in the muscle tissue of normal mice (data not shown). As expected, although animals in all treatment groups have similar body weight gain throughout the study (Additional file 1: Figure S4a), *Upf1*-ASO-treated mice showed statistically significant elevations of liver and spleen weights, and plasma AST levels; and *Smg6*-ASO-treated mice showed increases in liver weights and plasma ALT and AST levels (Additional file 1: Figure S4b and c). In contrast, the *Upf3b*-ASO was very well tolerated in mdx mice (Additional file 1: Figure S4a–c). By qPCR analysis, we found that ASO treatments reduced *Upf3b* and *Smg6* mRNA levels in tibialis anterior (TA) muscle by > 90% and 60%, respectively, compared to vehicle-treated controls (Fig. 4a and b), and *Upf1* mRNA by approximately 50% compared to controls (Fig. 4c). As expected, the approximately 50% reduction of *Upf1* did not affect dystrophin mRNA levels, but the ASO-mediated *Smg6* depletion upregulated dystrophin PTC-containing mRNAs by threefold compared to animals treated with DPBS and control-ASO (Fig. 4d). Interestingly, ASO-mediated downregulation of the modest NMD factor *Upf3b* also stabilized the dystrophin PTC-containing mRNAs to a comparable extent as the *Smg6*-ASO (Fig. 4d). These results suggested that ASO-mediated depletion of a modest NMD regulator could achieve a similar degree of NMD inhibition on a specific disease associated PTC-containing transcript, while simultaneously remaining more tolerable than the depletion of a robust NMD regulator.

We did not detect full-length dystrophin protein in either *Upf3b*-ASO or *Smg6*-ASO treated mice (Additional file 1: Figure S4D). It is likely that the low level of the basal translational read-through that occurs is insufficient to produce detectable amounts of the full-length proteins in this mouse model. A combination of a read-through agent with ASOs targeting NMD factors might result in full-length protein production. However, in our hands, the small-molecule read-through drugs gentamicin and PTC124 [45, 46] had no effect in this model (data not shown), so we were unable to test this hypothesis.

### *Upf3b*-ASO treatment stabilizes *hF9_R29X* mRNA and has minimum impact on the normal liver transcriptome in a mouse model of hemophilia B

Next, we tested the effect of the *Upf3b*-ASO in a hemophilia B mouse model. Hemophilia B is an X-linked bleeding disorder that results from a defect in the gene encoding coagulation factor IX (FIX), a serine protease that is critical for blood clotting [47]. Persons with severe hemophilia B have functional FIX levels that are < 1% of normal values and have frequent bleeding episodes that are associated with crippling arthropathy and early death [48]. Current treatment involves frequent intravenous injections of FIX protein concentrate; however, this treatment is prophylactic rather than curative and is associated with inhibitor formation [49]. A rise in circulating FIX to 1% of normal levels can substantially ameliorate the bleeding phenotype [50]. In this hemophilia mouse model, a human *FIX* minigene with a nonsense mutation (*hFIX-R29X*) is expressed in mice that lack the endogenous mouse *FIX* gene [51]. These mice recapitulate the phenotype of patients carrying the R29X mutation (CGA to TGA) who have severe hemophilia with no detectable circulating FIX protein [51]. The *hFIX* transgenes are driven by the human *transthyretin* promoter and thus are primarily expressed in mouse hepatocytes, which are the main cell type for endogenous factor IX production [51]. The mRNA expressed from the *hFIX-R29X* minigene was shown to be degraded by the NMD pathway in HepG2 cells [52]. The level of *hFIX-R29X* mRNA in these hemophilia mice is approximately 10% of the level of *hFIX* mRNAs in control mice that express a WT *hFIX* minigene [51, 52].

To improve ASO delivery to hepatocytes and minimize NMD inhibition in other cell types, we used an *Upf3b*-ASO conjugated to triantennary *N*-acetyl galactosamine (GalNAc). GalNAc is a high-affinity ligand for the hepatocyte-specific asialoglycoprotein receptor (ASGPR) [53–55]. GalNAc-conjugation results in enhanced ASO delivery to hepatocytes relative to non-parenchymal cells and potency that is six- to tenfold higher in mouse liver than an ASO of the same sequence without the GalNAc conjugation [56]. Due to these altered pharmacokinetic and dynamic properties, we first optimized the dosing regimen for the GalNAc-conjugated *Upf3b*-ASO (*Upf3b*-GalNAc-ASO) in normal mice. Normal mice were dosed every five days with a total of six doses of parent ASOs at previously optimized concentrations, or with five- or tenfold lower concentrations of the *Upf3b*-GalNAc-ASO. We found that the *Upf3b*-GalNAc-ASO had at least fivefold higher potency than the parent ASO, and was well tolerated (Additional file 1: Figure S5). Similar to the *Upf3b*-GalNAc-ASO, an *Upf1*-GalNAc-ASO had more than fivefold higher potency than the parent ASO (Additional file 1: Figure S5D). Moreover, the *Upf1*-GalNAc-ASO was better tolerated than the parent *Upf1*-ASO in mouse. Treatment with the parent *Upf1*-ASO at 50 mg/kg led to less body weight gain than observed in controls, whereas mice treated with 10 mg/kg *Upf1*-GalNAc-ASO, which results in better *Upf1* depletion, had weights similar to controls over the time course of the experiment (Additional file 1: Figure S5).

**Fig. 5:**
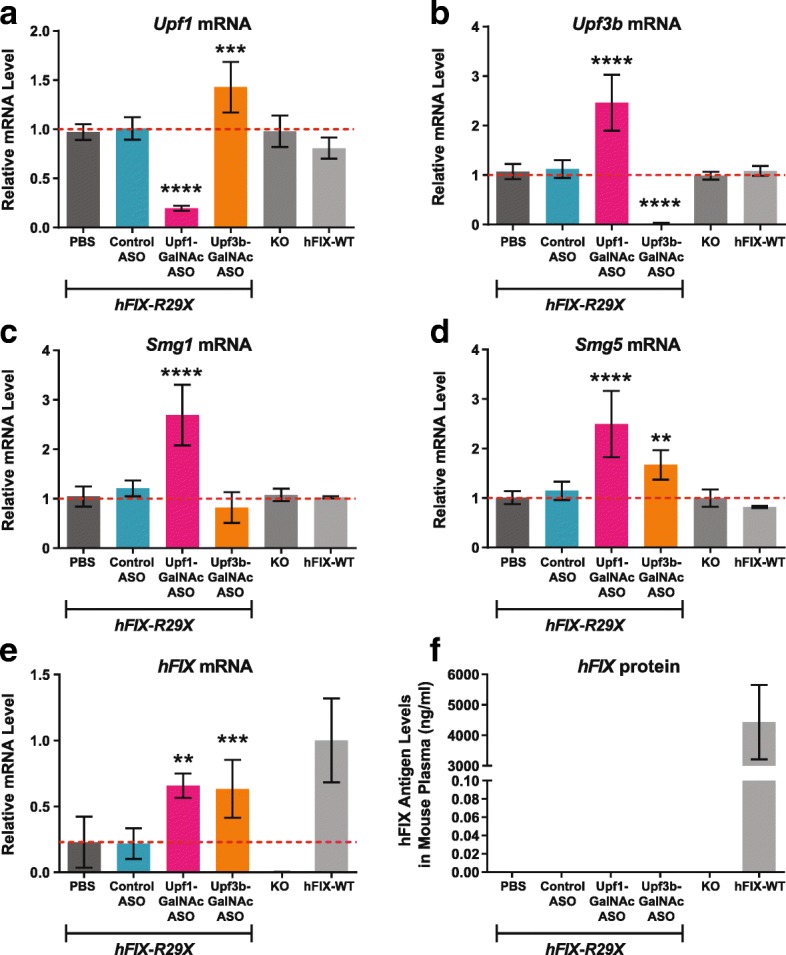
ASO-mediated *Upf3b* depletion stabilizes *hFIX-R29X* mRNA in hemophilia mice. *hFIX-R29X* mice aged 8–14 weeks (n = 5–6, 2–3 female and 2–3 male mice per group) were treated every five days with six total doses of DPBS, Control-GalNAc-ASO (15 mg/kg), *Upf1*-GalNAc-ASO (10 mg/kg), or *Upf3b*-GalNAc-ASO (10 mg/kg). Animals were sacrificed 48 h after the last dose. Untreated *FIX* knockout mice (KO) and *hFIX-WT* mice were used as controls. Results are presented as means ± standard errors. **a**–**d** mRNA levels of the indicated NMD factors that are also endogenous NMD substrates were analyzed by qPCR from mouse liver total RNA samples. *Gapdh* was used as an endogenous control. The expression levels in DPBS-treated mouse liver were set as 1. **e** *hFIX* mRNA levels measured by qPCR. *Gapdh* was used as an endogenous control. *hFIX* mRNA level in *hFIX-WT* mouse liver was set as 1. **f** Mouse plasma hFIX protein levels as measure by ELISA. Statistical significance was determined using a one-way ANOVA and Dunnett’s multiple comparison test in Prism. All groups were compared to DPBS-treated *hFIX-R29X* group. ** *p* < 0.01; *** *p* < 0.001; **** *p* < 0.0001

We next treated *hFIX-R29X* mice with GalNAc-conjugated ASOs targeting *Upf3b* and *Upf1*. The *Upf3b*-GalNAc-ASO was well tolerated in *hFIX-R29X* mice with a slight increase in liver weight, whereas *Upf1*-GalNAc-ASO treatment led to a modest increase in ALT/AST levels and elevations in liver weight (Additional file 1: Figure S6). *Upf1* mRNA levels were reduced by 80% and *Upf3b* by > 95% in liver (Fig. 5a and b). The depletion of *Upf1* significantly inhibited the NMD pathway as shown by upregulation of the endogenous NMD substrates including *Upf3b*, *Smg1*, and *Smg5* (Fig. 5b–d) and led to significant upregulation of *hFIX-29X* mRNA relative to the vehicle-treated controls (Fig. 5e). Interestingly, although the depletion of *Upf3b* resulted in moderate or no effects on the endogenous NMD substrates measured (Fig. 5a, c, and d), it significantly upregulated the level of *hFIX-R29X* mRNA to the level similarly induced by *Upf1*-ASO treatment (Fig. 5e). This suggests that the degradation of *hFIX-R29X* mRNA by NMD is executed by the UPF3B-dependent branch of the NMD pathway. ASO-mediated *Upf3b* depletion is sufficient to stabilize *hFIX-R29X* mRNA. As with the mdx mice, we did not detect hFIX protein in the plasma of mice treated with *Upf3b*-GalNAc-ASO (Fig. 5f).

**Fig. 6:**
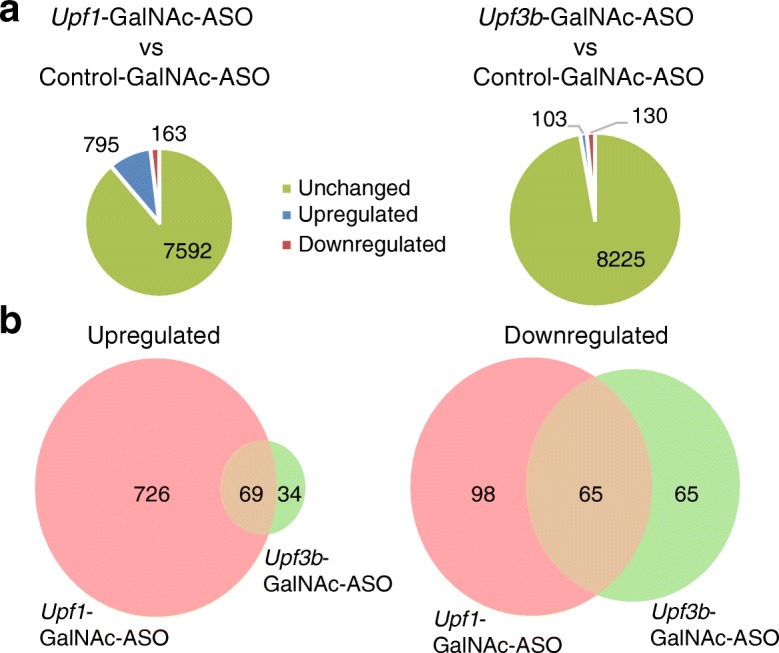
GalNAc-ASO-mediated *Upf3b* depletion in hemophilia mouse liver affects a small subset of normal transcripts. *hFIX-R29X* mice aged 8–14 weeks (*n* = 3–4) were treated every five days with six total doses of DPBS, Control-GalNAc-ASO (15 mg/kg), *Upf1*-GalNAc-ASO (10 mg/kg), or *Upf3b*-GalNAc-ASO (10 mg/kg). Animals were sacrificed 48 h after the last dose. Total RNA from livers was isolated and analyzed by transcriptome analysis. **a** *Pie charts* showing the numbers of differentially expressed genes in livers of *Upf1*-GalNAc-ASO treated mice compared to Control-GalNAc-ASO treated mice (*left*) and in livers of *Upf3b*-GalNAc-ASO treated mice compared to Control-GalNAc-ASO treated mice (*right*). **b** *Venn diagrams* comparing differentially expressed genes in livers of *Upf1*-GalNAc-ASO treated mice and *Upf3b*-GalNAc-ASO treated mice. *Left*: Upregulated genes. *Right*: Downregulated genes

In order to evaluate the impact of ASO-mediated *Upf3b* depletion on the normal transcriptome, we performed whole transcriptome analysis [57] on *hFIX-R29X* mouse livers treated with *Upf3b*-GalNAc-ASO. A scrambled GalNAc-ASO was used as a control. Livers from *Upf1*-GalNAc-ASO treated mice were also included to evaluate the impact on the normal transcriptome by ASO-mediated depletion of a robust NMD regulator. Treatment with the *Upf1*-GalNAc-ASO resulted in significant changes in levels of 958 transcripts relative to levels in control-GalNAc-ASO treated samples (level change ≥ 2-fold, *p* ≤ 0.01) among approximately 8400 genes quantified (transcripts per million [TPM] reads ≥ 5). Thus, about 11.4% of the mouse liver transcriptome was affected (Fig. 6a, left panel). Among these differentially expressed genes, 795 mRNAs were significantly upregulated and 163 mRNAs were significantly downregulated (Fig. 6a, left panel). In *Upf3b*-GalNAc-ASO treated *hFIX-R29X* mouse liver, only 233 mRNAs (2.8% of the mouse liver transcriptome) were significantly changed (Fig. 6a, right panel). Of these, 103 mRNAs were upregulated and 130 mRNAs were downregulated (Fig. 6a, right panel). Of the 103 mRNAs that were upregulated upon *Upf3b*-GalNAc-ASO treatment, 69 were also significantly upregulated in *Upf1*-GalNAc-ASO treated mouse liver (Fig. 6b, left panel). These mRNAs are probably endogenous NMD substrates that are regulated by a UPF3B-dependent NMD pathway. Only 65 transcripts were downregulated in both *Upf3b*-GalNAc-ASO and *Upf1*-GalNAc-ASO treated samples (Fig. 6b, right panel); these likely represent downstream effects of modulating endogenous NMD substrates. The regulated genes are listed in Additional file 2: Table S2. We performed KEGG pathway analysis on these differentially expressed genes to investigate if any biological pathway is significantly affected by either *Upf1*- or *Upf3b*-GalNAc-ASO mediated NMD inhibition. In *Upf1*-GalNAc-ASO treated samples, genes involved in drug metabolism, inflammation, DNA replication, and immune responses were significantly enriched (Additional file 2: Table S3). Thus, these analyses reflected changes in multiple biological pathways in *Upf1*-GalNAc-ASO treated livers, which is supported by the changes in tolerability measurements. On the contrary, in *Upf3b*-GalNAc-ASO treated samples, only genes involved in drug metabolism pathways were significantly enriched (Additional file 2: Table S3), which is consistent with the better tolerability profile of *Upf3b*-GalNAc-ASO treatment. These data indicated that ASO-mediated depletion of *Upf3b* in mouse liver induced very few changes to the normal transcriptome compared to the depletion of the robust NMD regulator *Upf1*. This result was consistent with observations in human HeLa cells [28], and supports that UPF3B protein only regulates a subset of endogenous NMD substrates.

### A *Upf3b*-GalNAc-ASO in combination with translational read-through molecules improved FIX coagulation activity in hemophilia B mice

To evaluate if the inhibition of the NMD pathway by *Upf3b*-GalNAc-ASO improves the efficacy of read-through therapy, we tested combination treatments of *Upf3b*-GalNAc-ASO and read-through agents in hemophilia B mice. Previously, treatment of *hFIX-R29X* mice with the small-molecule read-through drug geneticin alone did not result in detectable full-length protein in plasma [52]. This could be because: (1) the *hFIX-R29X* mRNA level was insufficient for translational read-though to produce detectable amounts of full-length protein; and (2) this small molecule read-through drug is not efficient in promoting translational read-through of *hFIX-R29X* mRNA. Since the depletion of the translation termination factor eRF3a leads to efficient translational read-through of a reporter gene in human cells [58], we developed an ASO targeting *Gspt1* mRNA, the mouse homolog of *eRF3a*, as a second approach in addition to geneticin to promote efficient read-through in vivo. In order to maximize the read-through efficiency, we also tested the triple combination of *Upf3b*-GalNAc-ASO, *Gspt1*-GalNAc-ASO, and geneticin.

*hFIX-R29X* mice were treated with *Upf3b*-GalNAc-ASO (10 mg/kg), *Gspt1*-GalNAc-ASO (5 mg/kg) or a combination of *Upf3b*-GalNAc-ASO (10 mg/kg) and *Gspt1*-GalNAc-ASO (5 mg/kg) every five days with six total doses for 4.5 weeks. During the last week of treatment, one group of each *Upf3b*-GalNAc-ASO-treated, *Gspt1*-GalNAc-ASO-treated or *Upf3b*-GalNAc-ASO/Gspt1-GalNAc-ASO-treated mice were also treated with 28 mg/kg geneticin daily for seven days. Animals were sacrificed 48 h after the last ASO treatments and 9 h after the last dose of geneticin.

**Fig. 7:**
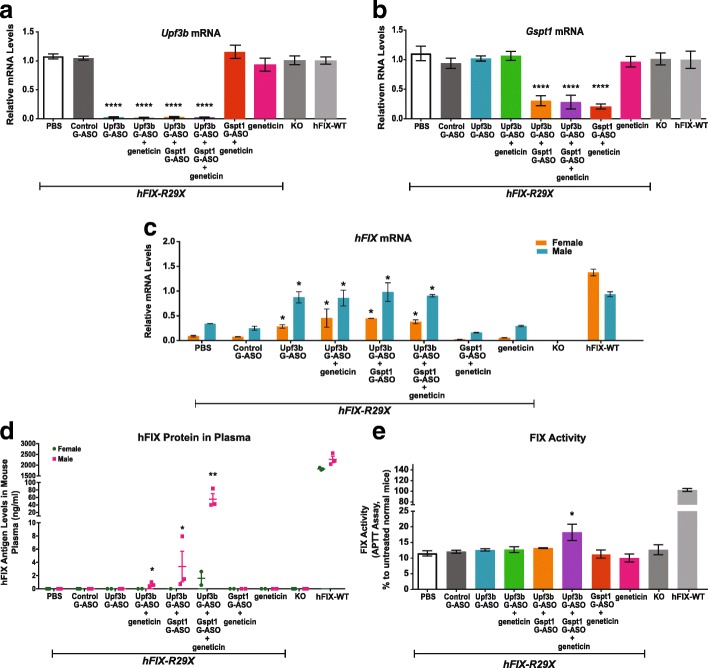
Combination treatment of *Upf3b*-ASO and read-through agents results in production of full-length hFIX protein and improved coagulation activity in *hFIX*-R29X mice. *hFIX-R29X* mice aged 8–14 weeks (n = 5–6, 2–3 female and 3 male mice per group) were treated every five days with six total doses of DPBS, Control-GalNAc-ASO (Control G-ASO, 15 mg/kg) *Upf3b*-GalNAc-ASO (Upf3b G-ASO, 10 mg/kg), *Gspt1*-GalNAc-ASO (Gspt1 G-ASO, 5 mg/kg), or a combination of *Upf3b*-GalNAc-ASO (Upf3b G-ASO, 10 mg/kg) and *Gspt1*-GalNAc-ASO (Gspt1 G-ASO, 5 mg/kg). Geneticin (28 mg/kg) was administered daily during the final seven days of the study either alone, in combination with *Upf3b*-GalNAc-ASO treatment, *Gspt1*-GalNac-ASO treatment, or in combination with *Upf3b*- and *Gspt1*-GalNAc-ASOs as described. Animals were sacrificed 48 h after the last dose of ASO and 9 h after the last dose of geneticin. Untreated KO and *hFIX*-WT mice were used as controls. Results are presented as means ± standard errors. **a** *Upf3b*, **b** *Gspt1*, and **c** *hFIX* mRNA levels were analyzed by qPCR from mouse liver total RNA samples. *Gapdh* was used as an endogenous control. The expression levels in *hFIX-WT* mouse liver were set as 1. **d** Mouse plasma hFIX protein levels as measure by ELISA. **e** APTT FIX activity assay in plasma from treated male mice (n = 3). Data were normalized using a standard curve generated with pooled WT C57BL6/J mouse plasma. Statistical significance was determined using either a one-way ANOVA (**a**, **b**, **e**) or a two-way ANOVA (**c**, **d**) and Dunnett’s multiple comparison test in Prism. All groups were compared to DPBS-treated *hFIX-R29X* mouse group. * *p* < 0.05; ** *p* < 0.01; **** *p* < 0.0001

The expression of both *Upf3b* and *Gspt1* were significantly reduced by ASO treatments (Fig. 7a and b, and Additional file 1: Figure S7). ASO-mediated depletion of *Upf3b* in mouse livers led to significant upregulation of *hFIX-R29X* mRNA (Fig. 7c). Of note, we observed differential *hFIX-R29X* mRNA levels in male and female *hFIX-R29X* mice (Fig. 7c), which was also observed in our previous experiment (Fig. 5), but has not been reported previously. In these mice, *hFIX-R29X* mRNA expression was 4.7-fold higher in male than in female mice (Fig. 7c). When treated with the *Upf3b*-GalNAc-ASO, the level of *hFIX-R29X* mRNA in male mice increased from approximately 30% to 93% of the level in male *hFIX-WT* mice (approximately a threefold upregulation) (Fig. 7c). In female *hFIX-R29X* mice, *hFIX* mRNA was basally expressed about 7.8% of levels in *hFIX-WT* mice. *hFIX-R29X* mRNA levels were upregulated to about 34% of levels in *hFIX-WT* mice after *Upf3b*-GalNAc-ASO treatment (approximately a fourfold upregulation), which was still significantly lower than male mice treated with *Upf3b*-GalNAc-ASO (Fig. 7c). The reason for the *hFIX-R29X* differential expression in male and female mice is unclear. Interestingly, when *Upf3b*-GalNAc-ASO treatment was combined with geneticin or *Gspt1*-ASO treatment, we detected hFIX protein in male mouse plasma but not in plasma from female mice (Fig. 7d). The level of *hFIX-R29X* mRNA in female hemophilia mice may not have been sufficient to produce detectable full-length protein in the presence of read-through agents. The amount of hFIX protein detected in male hemophilia mice with either the combination of *Upf3b*-GalNAc-ASO and *Gspt1*-GalNAc-ASO, or the combination of *Upf3b*-GalNAc-ASO and geneticin, although reliably detected, remained lower than the threshold 1% of *hFIX-WT* abundance (Fig. 7d). Interestingly, when combining all three treatments together (*Upf3b*-GalNAc-ASO/*Gspt1*-GalNAc ASO/geneticn), we were able to detect full-length hFIX proteins in female hemophilia mice, while the abundance was < 1% of *hFIX-WT* mice. Moreover, in the male mice received triple combination treatment, the plasma full-length hFIX protein level rose to ~ 2–3% of the level in *hFIX-WT* animals (Fig. 7d). No full-length hFIX protein was detected in mice with combination treatment of *Gspt1*-GalNAc-ASO and geneticin, indicating the stabilization of *hF9-R29X* mRNA by NMD-inhibition is critical in producing full-length protein in this mouse model. To determine if the increased production of full-length hFIX protein in male *hFIX-R29X* mice resulted in an enhanced FIX activity, activated partial thromboplastin time (APTT) was determined in samples from untreated male mice and male mice treated with *Upf3b*-GalNAc-ASO, geneticin, *Gspt1*-GalNAc-ASO or combinations of these agents. As expected, the triple combination treatment, but not treatments with individual or double combinations, led to significant coagulation activity rescue in male hemophilia mice (Fig. 7e). In summary, we showed that GalNAc-ASO-mediated depletion of *Upf3b* in *hFIX-R29X* mice was well tolerated, significantly stabilized the level of the *hFIX-R29X* mRNA, and only affected a small subset of normal transcripts. We further showed that combining *Upf3b*-GalNAc-ASO treatment with reagents that induce translational read-through led to the production of full-length hFIX protein and improvement of hFIX coagulation activity.

## Discussion

In this study, we generated a catalog of efficacious and safe antisense oligonucleotides targeting each component of the NMD pathway. Testing these ASOs in vitro, we classified NMD factors into three categories: “robust,” “modest,” and “passive” based on their regulation of NMD. ASOs targeting the robust regulators *Upf1*, *Upf2*, *Smg1*, and *Smg6* efficiently inhibited degradation of a reporter-gene-based NMD target and regulated most endogenous NMD substrates tested (Figs. 1 and 2). ASOs targeting the modest NMD regulators *Upf3b*, *Smg5*, and *Smg7* only moderately affected the reporter and upregulated a few endogenous NMD substrates tested (Figs. 1 and 2). ASOs targeting the passive NMD regulators *Smg8* and *Smg9* did not alter reporter degradation and stabilized few endogenous NMD substrates (Figs. 1 and 2). In line with this, ASO-mediated depletion of *Smg8* and *Smg9* in mouse liver were well tolerated (Additional file 2: Table S1). Also, as expected, ASO-mediated depletion of the robust NMD regulators were less well tolerated compared to the depletion of the modest NMD regulators in vivo (Additional file 2: Table S1). These results are consistent with reports that the complete knock out of robust NMD regulators *Upf1*, *Upf2*, *Smg1*, and *Smg6* result in early embryonic lethality in mice [21–24], whereas *Upf3b*-null mice are viable and fertile [38, 59].

Interestingly, depletion of the robust NMD factor *Smg6* was better tolerated than the depletion of other robust NMD factors. SMG6 mediates endonucleolytic degradation of PTC-containing transcripts, which may also be degraded by the SMG5-SMG7 mediated exonucleolytic decay pathway. Recently, a transcriptome analysis revealed that SMG6 and SMG7 act on essentially the same transcripts, indicating extensive redundancy between the endo- and exonucleolytic decay routes [30]. These results may underlie the tolerability of ASO-mediated *Smg6* depletion in vivo. Both in vitro (Figs. 1 and 2) and in vivo (data not shown) results indicate that ASO-mediated *Smg6* depletion induced a much more robust NMD inhibition compared to either *Smg5* or *Smg7* depletion. This suggests that, at least in liver cells, the SMG6-mediated endonucleolytic decay pathway could be the primary decay pathway for NMD and that the SMG5-SMG7-mediated exonucleolytic decay pathway could function as a secondary decay mechanism. In support of this, ASOs targeting *Smg5* and *Smg7* are much better tolerated in mouse liver than the *Smg6*-ASO (Additional file 2: Table S1).

The depletion of the third modest NMD regulator *Upf3b* to > 95% was well tolerated in both normal and diseased mice. This is probably because UPF3B only regulates a subset of the NMD substrates, as it has been reported that NMD can occur in the absence of UPF3B [28, 38, 59]. Using whole transcriptome analysis of mouse livers treated with ASOs, we confirmed that only ~ 2.8% of detected transcripts are affected by ASO-mediated *Upf3b* depletion, which is a very small subset compared to the ~ 11.4% of the liver transcriptome affected by ASO-mediated *Upf1* depletion (Fig. 6).

To our surprise, the depletion of *Upf3b* elicited significant upregulation of the PTC-containing mutant mRNAs to levels equivalent to 60–70% of WT transcript expression in both mdx and hemophilia mouse models (Figs. 4 and 5). UPF3B levels increase during myogenesis, resulting in an increase in the efficiency of UPF3B-dependent NMD [60]. This could contribute to the sensitivity of *Upf3b* depletion in the muscles of mdx mice. In the *hFIX-R29X* mice, the nonsense codon is located in the last exon of the minigene. This transcript, due to a lack of exon junctions downstream of the PTC, should be subjected to EJC-independent NMD. UPF3B protein was originally thought to function as a bridge between the EJC and UPF1. Recently, it has become clear that UPF3B also functions in the EJC-independent NMD pathway. In the absence of a downstream EJC, the recruitment of UPF3B to PTC-containing mRNAs occurs slowly, and therefore these PTC-containing transcripts are more sensitive to the depletion of UPF3B [61]. Overall, our results suggested that the UPF3B-dependent NMD pathway plays an important role in degrading PTC-containing mutant transcripts in both mdx and hemophilia mouse models, which seems to be in accordance with a recent report that assigned a central role for UPF3B in the NMD pathway [62]. Using a fully reconstituted in vitro translation system, Neu-Yilik et al. demonstrated that UPF3B plays dual role in both early and late translation termination and therefore is involved in the crosstalk between NMD machinery and the PTC-bound ribosome, a central mechanistic step of NMD [62]. However, this raises the question of why the UPF3B-dependent NMD branch appears to regulate a small subset of endogenous substrates. The answer might lie in the fundamental differences in translation termination between PTCs in mutant transcripts and NMD-induceing stop codons in  normal transcripts. 

Although it significantly increased the PTC-containing transcript levels, *Upf3b*-ASO treatment alone did not ameliorate disease phenotypes in either mdx or hemophilia mouse model. This is likely due to two reasons: first, the truncated proteins produced in these two mouse models have no residual function; and second, the basal read-through levels are low for both of the transcripts. Importantly, *Upf3b*-ASO-mediated NMD inhibition did significantly improve the efficacy of the small molecule read-through drug geneticin (Fig. 7). Furthermore, when combined with *Gspt1*-ASO treatment to further promote translational read-through, plasma hFIX protein level rose to ~ 2–3% of that in the WT mice (Fig. 7d). The increased hFIX protein was functional, as shown by significant improvement in hFIX activity in plasma from mice treated with the triple combination of *Upf3b*-ASO, *Gspt1*-ASO, and geneticin (Fig. 7e). Therefore, inhibition of NMD with *Upf3b*-ASO could be part of a viable therapeutic approach for diseases caused by this type of nonsense mutation. In future studies, it will be important to evaluate the therapeutic effect of ASO-mediated *Upf3b* depletion as a standalone treatment in a disease model where the mutant protein remains at least partially functional.

Of note, NMD is a cell type- and tissue-specific process [63]. Different cell types differ in their sensitivity to NMD factor depletion [63]. For example, *Smg8* was shown to be essential for the degradation of a PTC-containing mRNA encoding a mutant collagen in fibroblasts isolated from patients with Ullrich congenital muscular dystrophy [13]. In this system, siRNAs targeting *Smg8* restored defective mRNA and protein levels without affecting cell growth [13]. Therefore, it will be important to test the effects of depleting each NMD factor in relevant models in vivo to identify the optimal therapeutic target. The ASOs we generated provide tools to enable these experiments in murine systems.

## Conclusions

In this study, we took advantage of antisense technology to systematically target each component of the nonsense-mediated mRNA decay pathway to investigate whether NMD inhibition has the potential as a therapeutic strategy for treating human diseases caused by nonsense mutations. We demonstrated that depletion of the modest NMD regulator *Upf3b* was well tolerated in both normal and diseased mice. Importantly, we showed that ASO-mediated depletion of *Upf3b* significantly stabilized PTC-containing mutant transcripts and had a minimal impact on the normal transcriptome. Further, we demonstrated that *Upf3b*-ASO treatment significantly enhanced the efficacy of read-through therapy and led to improved coagulation activity in a hemophilia mouse model. Our results suggest that ASO-mediated depletion of the NMD factor UPF3B is potentially a safe and effective approach for the treatment of diseases caused by nonsense mutations.

## Methods

### Antisense oligonucleotides

Antisense oligonucleotides used in this study were chemically modified with phosphorothioate in the backbone and constrained ethyl (cET) modifications in the wings with a central 10-nucleotide deoxy gap (3-10-3 gapmer). Oligonucleotides were synthesized using an Applied Biosystems 380B automated DNA synthesizer (PerkinElmer Life and Analytical Sciences) and purified as previously described [34, 64]. ASO sequences are provided in Additional file 2: Table S1. Lyophilized ASOs were dissolved in sterile DPBS (without calcium or magnesium) and quantified by ultraviolet spectrometry, diluted to the desired concentration, sterilized through a 0.2-μm filter.

### Cell culture and transfection

Mouse liver MHT cells [65] were cultured in DMEM containing 10% fetal bovine serum, in 5% CO_2_ at 37 °C. Cells were co-transfected with WT or mutant β-GLOBIN-luciferase construct encoding the Renilla luciferase with a Firefly luciferase control construct [36] using Effectene transfection reagent following the manufacturer’s protocol (Qiagen). Transfected cells were then selected by G418 and single colonies were isolated for further analysis. For ASO treatment, stably transfected cells were seeded at 96-well plate at concentration of 5000 cell/well. ASOs were added to the culture media 5–12 h after seeding cells at the indicated concentrations. Cells were harvested 72 h after ASO-treatment.

### Animals

All the animals of wild-type (purchased from JAX), mdx [66] (purchased from JAX), and hemophilia mice [51] (licensed from The Children’s Hospital of Philadelphia and maintained in Taconic) genotypes were housed under standard conditions in a pathogen-free mouse facility. All animal studies were approved by Institutional Animal Care and Use Committees at Ionis Pharmaceuticals and were conducted in accordance with the United States Public Health Service’s Policy on Human Care and Use of Laboratory Animals. ASOs were administrated subcutaneously at a volume of 10 μL/g. Geneticin (G418) (5.6 mg/mL) was administrated subcutaneously at a volume 5 μL/g.

### Plasma chemistry analysis

Blood samples were collected by cardiac puncture at time of sacrifice. Plasma chemistry values were measured on the AU480 Clinical Chemistry Analyzer (Beckman Coulter).

### RNA analysis

Cultured cells were lysed and the total RNA was extracted with Qiagen RNeasy columns. Animal tissues were homogenized in guanidine isothiocyanate solution (Invitrogen) supplemented with 8% 2-mercaptoethanol (Sigma-Aldrich). Total RNA was prepared using the RNeasy Mini Kit (Qiagen). Quantitative real-time PCR (qRT-PCR) was performed using an ABI step-one sequence detector. Taqman primer probe sequences are listed in Additional file 2: Table S4.

### Protein analysis

NMD factor protein levels were measured using western blot. Cultured cells were lysed with RIPA buffer (Thermo Fisher Scientific) containing Halt Protease Inhibitor Cocktail (Life Technologies). Animal tissues were homogenized in the same buffer. Protein concentrations were determined using the BioRad DC protein assay, and protein was loaded (25 ug for cell samples and 40 ug for tissue samples) onto a 4–15% Criterion™ TGX™ Precast Midi Protein Gel (BioRad). Western blot membranes were probed with primary anti-UPF1 antibody, anti-UPF2 antibody (both generously provided by Dr. Jens Lykke-Anderson (UCSD), anti-UPF3B antibody (Boster Immunoleader PB9843), anti-SMG1 antibody (Bethyl Laboratory A301-535A), anti-SMG6 antibody (Abcam ab87539), anti-SMG9 antibody (Abcam ab85659), or anti-GSPT1 antibody (Abcam ab49878). An antibody against β-Actin (Sigma A5316) was used as a loading control. Membranes were then incubated with IRDye secondary antibodies (Li-COR) and scanned using an Odyssey infrared system (Li-COR). Images were quantified using Image Studio (Li-COR). The western blot for dystrophin was performed using NuPAGE Novex 3–8% Tris-Acetate Protein Gel (Life Technologies). Blot was incubated with anti-Dystrophin (Leica NCL-DYS2; 1:100) and anti-alpha actinin (Abcam EP2529Y; 1:20,000) followed by anti-mouse IgG, HRP-linked antibody (CST#7076) anti-rabbit IgG, HRP-linked antibody (CST #7074) and then detected using the Amersham ECL Prime Western Blotting Detection Reagent (GE RPN2232 Life Sciences).

hFIX protein level in mouse plasma was measured by ELISA using Human Factor IX ELISA Kit (Abcam ab188393) following manufacture instructions.

### RNA-sequencing analysis

RNA-sequencing (RNA-seq) was performed by sequencing fragmented libraries from purified total RNA using Illumina Tru-seq protocol. Samples were sequenced on an Illumina HiSeq2500 average depth of approximately 30 × 10^6 reads and had an average alignment rate of 70%. Read data were demultiplexed and transcript quantitation was performed using Salmon-Ver-0.7.2 with the quasi-mapping based mode and default parameters (Automated libType detection: -l A) [67]. A Salmon gene model index was built using complementary DNA sequences from Ensembl Mus musculus Build-81 (genome build GRCm38) and used in read alignment. Expression values are reported as TPM. Genes were identified as differentially expressed assuming a model of gene expression variance derived from a negative binomial distribution and based upon gene expression levels in the reference cohorts. Gene-specific *p* values were computed for each biological replicate and median-aggregated. Genes whose median aggregate *p* value were ≤ 0.01 and had an average log2-fold-change magnitude > 1 within a group were considered significant and used in downstream analysis.

Differentially expressed genes were analyzed according to predefined pathways or functional categories annotated by KEGG [11] using the DAVID bioinformatic resource [29].

### FIX activity assay

FIX activity assay was done at UCSD Murine Hematology and Coagulation Core Laboratory. In brief, clotting times are determined in duplicate with an ST4 semi-automated mechanical coagulation instrument (Diagnostica Stago, NJ). A total of 30 μL of citrated sample plasma diluted 1/10 in HN/BSA buffer are incubated with 30 μL of APTT reagent and 30 μL of human citrated plasma deficient of factor IX at 37 °C for 5 min, followed by the addition of 30 μL of 25 mM 37 °C CaCl2 to initiate clotting. Time until clot formation is measured and interpolated on a standard curve of serial dilutions citrated normal (BL/6 pool) mouse plasma tested as described to give reported result in % BL/6.

## Additional files

Additional file 1: This file contains seven supplementary figures (**Figures S1–S7**). (PDF 679 kb)

Additional file 2: This file contains four supplementary tables (**Tables S1–S4**). (XLSX 95 kb)
