# Supplementary material for: Antisense suppression of the nonsense mediated decay factor Upf3b as a potential treatment for diseases caused by nonsense mutations
Source: Genome Biol. 2018 Jan 15;19:4. doi: 10.1186/s13059-017-1386-9 (PMC5769327; doi:10.1186/s13059-017-1386-9)
Supplement: Supplementary file 1 — This file contains seven supplementary figures (Figures S1–S7). (PDF 679 kb) [file 13059_2017_1386_MOESM1_ESM.pdf]

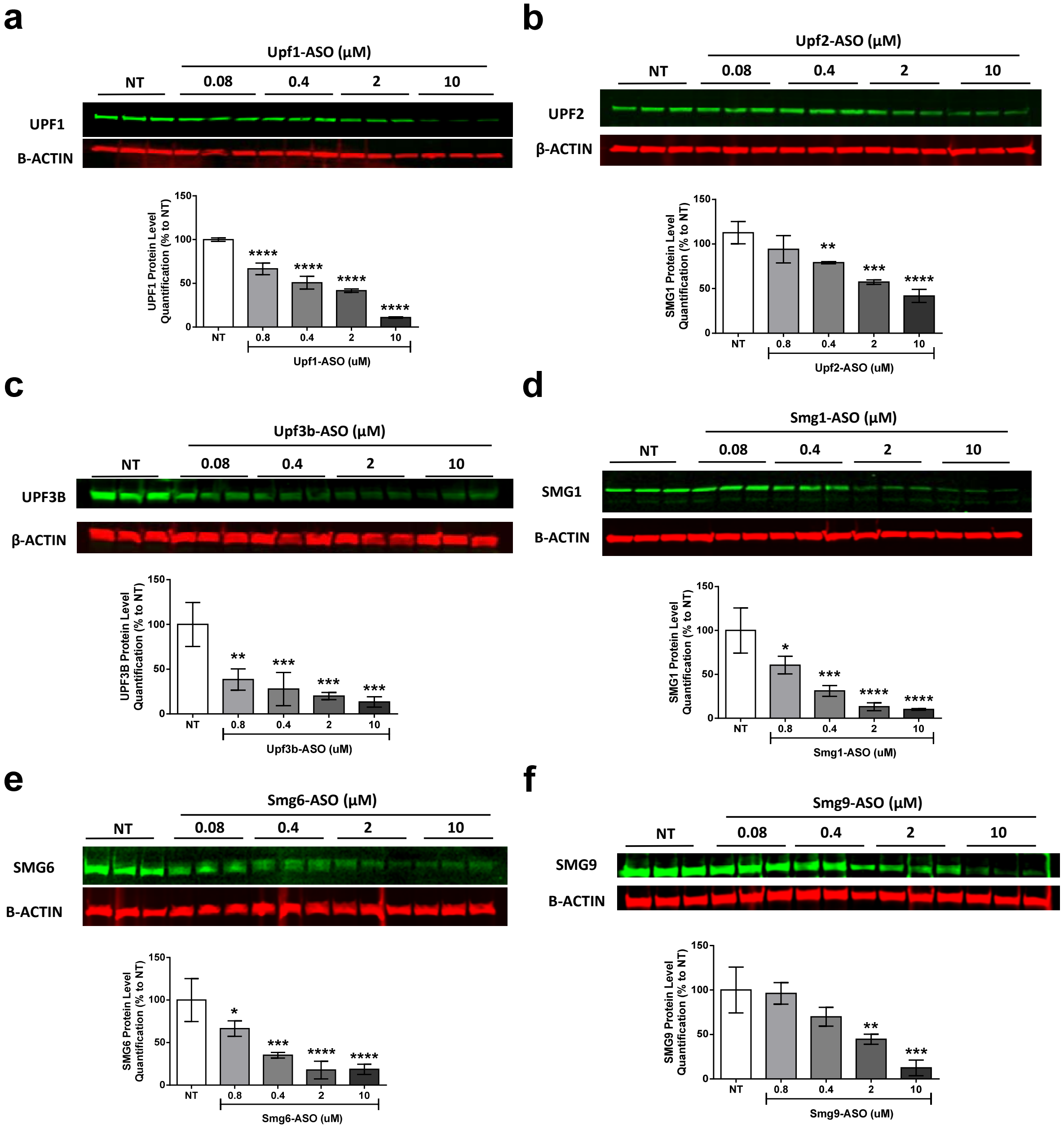
a
Upf1-ASO (μM)
NT
0.08
0.4
2
10
UPF1
B-ACTIN
UPF1 Protein Level Quantification (% to NT)
150
100
50
0
****
****
****
****
NT
0.8
0.4
2
10
Upf1-ASO (uM)
b
Upf2-ASO (μM)
NT
0.08
0.4
2
10
UPF2
β-ACTIN
SMG1 Protein Level Quantification (% to NT)
150
100
50
0
**
***
****
NT
0.8
0.4
2
10
Upf2-ASO (uM)
c
Upf3b-ASO (μM)
NT
0.08
0.4
2
10
UPF3B
β-ACTIN
UPF3B Protein Level Quantification (% to NT)
150
100
50
0
**
***
***
***
NT
0.8
0.4
2
10
Upf3b-ASO (uM)
d
Smg1-ASO (μM)
NT
0.08
0.4
2
10
SMG1
B-ACTIN
SMG1 Protein Level Quantification (% to NT)
150
100
50
0
*
***
****
****
NT
0.8
0.4
2
10
Smg1-ASO (uM)
e
Smg6-ASO (μM)
NT
0.08
0.4
2
10
SMG6
B-ACTIN
SMG6 Protein Level Quantification (% to NT)
150
100
50
0
*
***
****
****
NT
0.8
0.4
2
10
Smg6-ASO (uM)
f
Smg9-ASO (μM)
NT
0.08
0.4
2
10
SMG9
B-ACTIN
SMG9 Protein Level Quantification (% to NT)
150
100
50
0
**
***
NT
0.8
0.4
2
10
Smg9-ASO (uM)

Fig. S1

Figure S1. ASO targeting NMD factors achieve protein level target reduction in a dose-dependent manner

Mouse MHT cells were treated with ASOs targeting mouse NMD factors by free uptake for 72 hrs at the indicated concentrations. NMD factor protein levels were evaluated by western blot with antibodies specific to each of the NMD factors. β-Actin was included as loading control. Western blot membranes were scanned using an Odyssey imaging system and quantified using Image Studio (LI-COR). Quantification results are presented as means plus or minus standard errors (n=3). The protein level in NT samples was set as 1. **a** *Upf1*-ASO treated cells. **b** *Upf2*-ASO treated cells. **c** *Smg1*-ASO treated cells. **d** *Smg6*-ASO treated cells. **e** *Upf3b*-ASO treated cells. **f** *Smg9*-ASO treated cells. Statistical significance was determined using a one-way ANOVA and Dunnett's multiple comparison test in Prism. All groups were compared to NT group within each measurement. * $p<0.05$; ** $p<0.01$; *** $p<0.001$; **** $p<0.0001$.

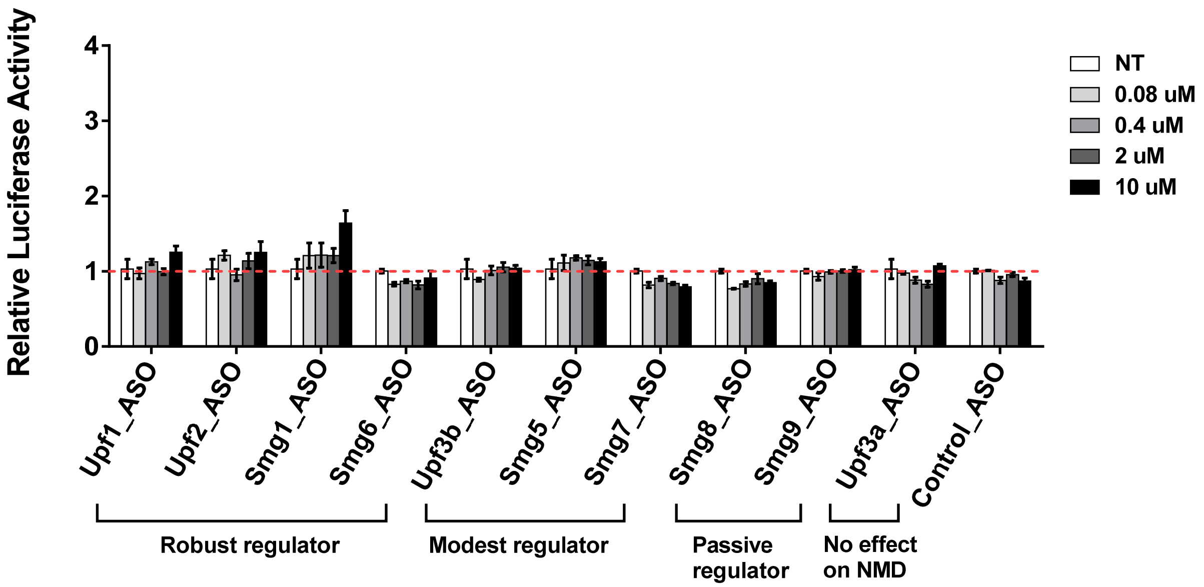
Relative Luciferase Activity
4
3
2
1
0
NT
0.08 uM
0.4 uM
2 uM
10 uM
Upf1_ASO
Upf2_ASO
Smg1_ASO
Smg6_ASO
Upf3b_ASO
Smg5_ASO
Smg7_ASO
Smg8_ASO
Smg9_ASO
Upf3a_ASO
Control_ASO
Robust regulator
Modest regulator
Passive regulator
No effect on NMD

Fig. S2

Figure S2. ASOs targeting NMD factors do not affect wild-type β-*GLOBIN* luciferase reporter.

Relative luciferase activity is shown from the wild-type β-*GLOBIN* luciferase reporter cell line after ASO treatment. Cells were treated with ASOs targeting the mouse NMD factors *Upf1*, *Upf2*, *Smg1*, *Smg6*, *Upf3b*, *Smg5*, *Smg7*, *Smg8*, *Smg9,* or *Upf3a* by free uptake at the concentrations indicated for 72 hrs. Results are normalized and presented as in Figure 1b.

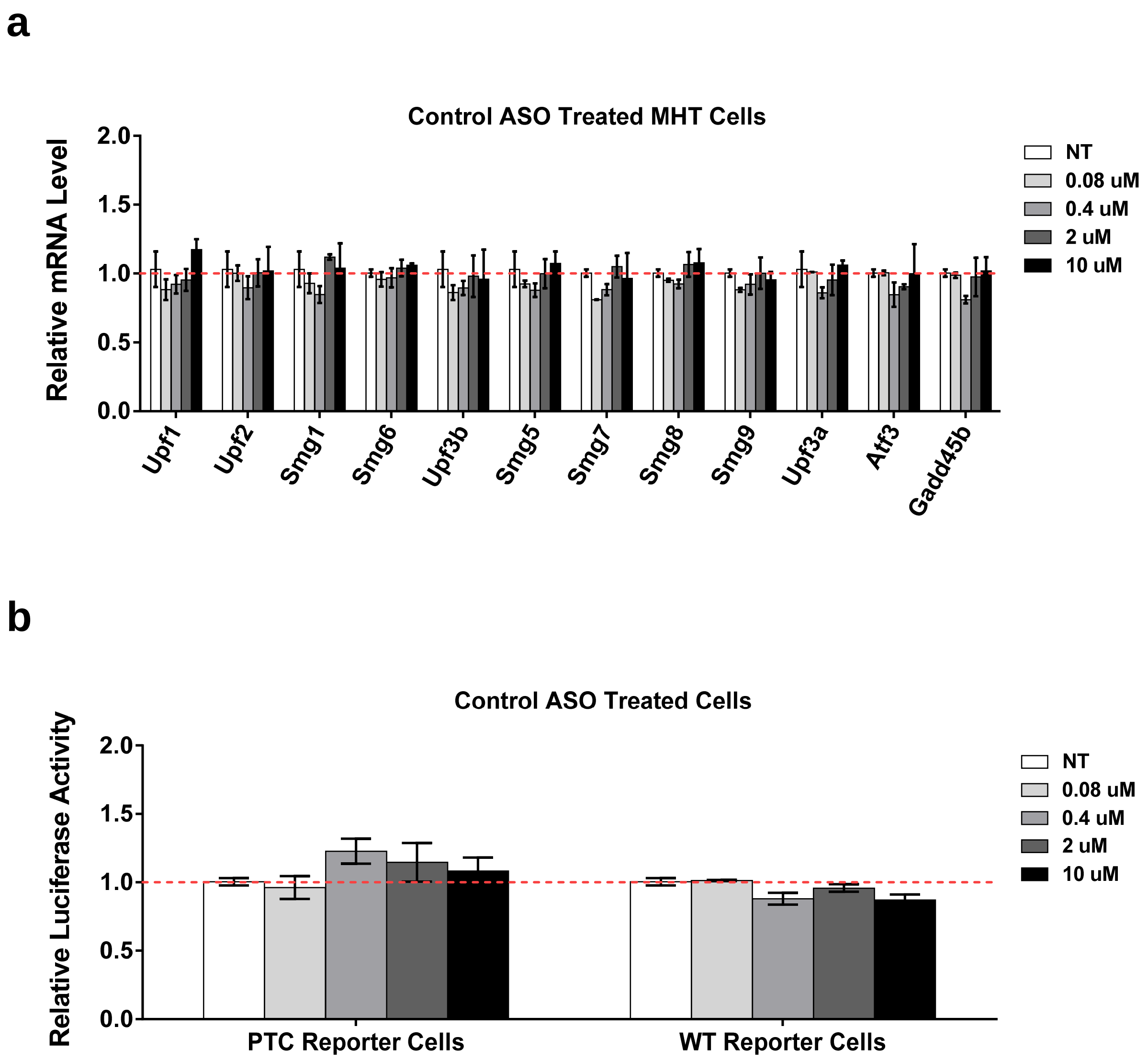
a
Control ASO Treated MHT Cells
Relative mRNA Level
2.0
1.5
1.0
0.5
0.0
Upf1
Upf2
Smg1
Smg6
Upf3b
Smg5
Smg7
Smg8
Smg9
Upf3a
Atf3
Gadd45b
NT
0.08 uM
0.4 uM
2 uM
10 uM
b
Control ASO Treated Cells
Relative Luciferase Activity
2.0
1.5
1.0
0.5
0.0
PTC Reporter Cells
WT Reporter Cells
NT
0.08 uM
0.4 uM
2 uM
10 uM

Fig. S3

Figure S3. Control ASO has no effect on the levels of the NMD factors and the NMD substrates.

Mouse MHT cells were treated with a control-ASO by free uptake for 72 hrs at the indicated concentrations. Results are normalized and presented as in Figure 1. **a** qPCR analysis of the mRNA levels of each NMD factor and/or NMD substrate. **b** Relative luciferase activity is shown from the PTC- or WT-β-*GLOBIN* luciferase reporter cell line.

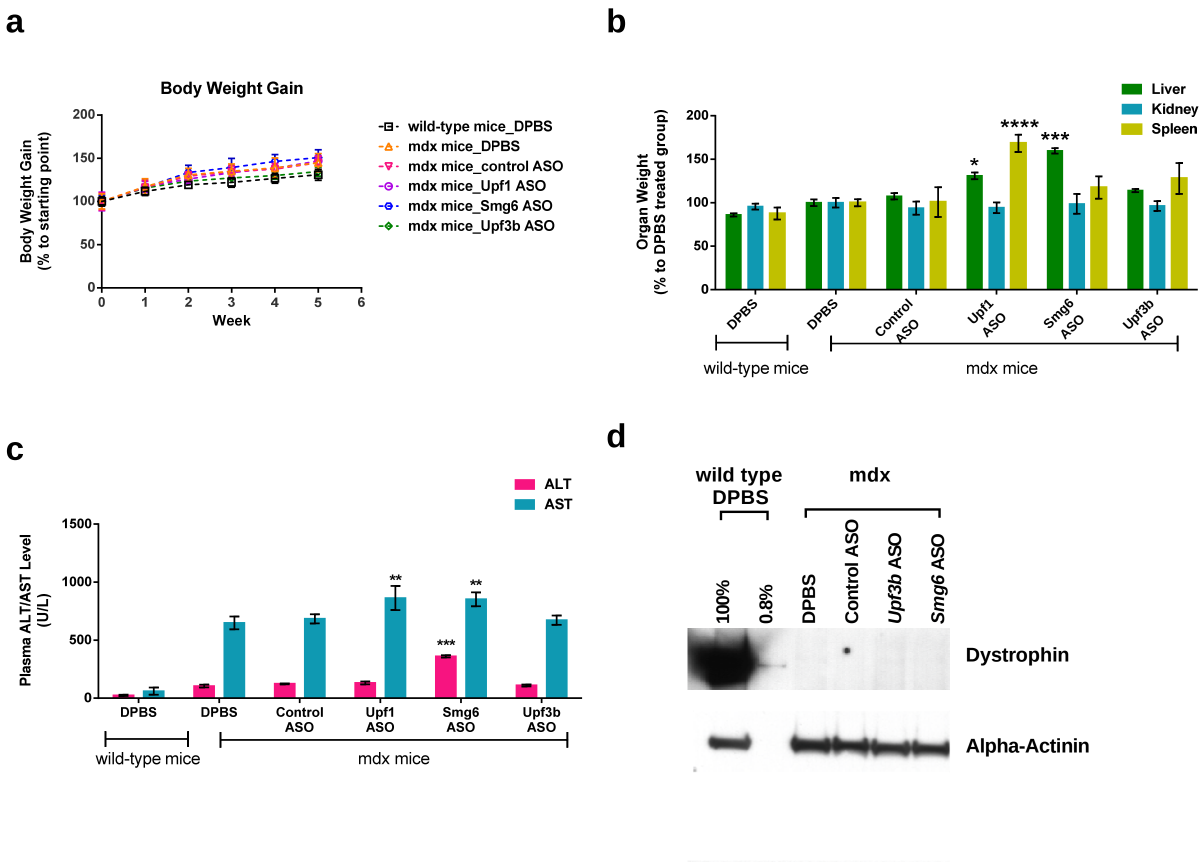
a
Body Weight Gain
Body Weight Gain (% to starting point)
Week
wild-type mice_DPBS
mdx mice_DPBS
mdx mice_control ASO
mdx mice_Upf1 ASO
mdx mice_Smg6 ASO
mdx mice_Upf3b ASO
b
Organ Weight (% to DPBS treated group)
Liver
Kidney
Spleen
DPBS
DPBS
Control ASO
Upf1 ASO
Smg6 ASO
Upf3b ASO
wild-type mice
mdx mice
c
Plasma ALT/AST Level (U/L)
ALT
AST
DPBS
DPBS
Control ASO
Upf1 ASO
Smg6 ASO
Upf3b ASO
wild-type mice
mdx mice
d
wild type DPBS
mdx
100%
0.8%
DPBS
Control ASO
Upf3b ASO
Smg6 ASO
Dystrophin
Alpha-Actinin

Fig. S4

Figure S4. ASO-mediated Upf3b depletion is well tolerated in mdx mice.

Study was performed as described in Figure 4. Results are presented as means plus or minus standard errors. **a** Body weights measured once a week. **b** Liver, kidney, and spleen weights measured at necropsy. **c** Plasma ALT and AST levels measured by clinical analyzer at necropsy. **d** Dystrophin protein levels from TA tissue samples analyzed by western blot with a dystrophin-specific antibody. Alpha-actinin was included as a loading control. Statistical significance was determined using a one-way ANOVA and Dunnett's multiple comparison test in Prism. All groups were compared to DPBS treated mdx mouse group within each measurement. **p<0.01; *** p<0.001; ****p<0.0001.

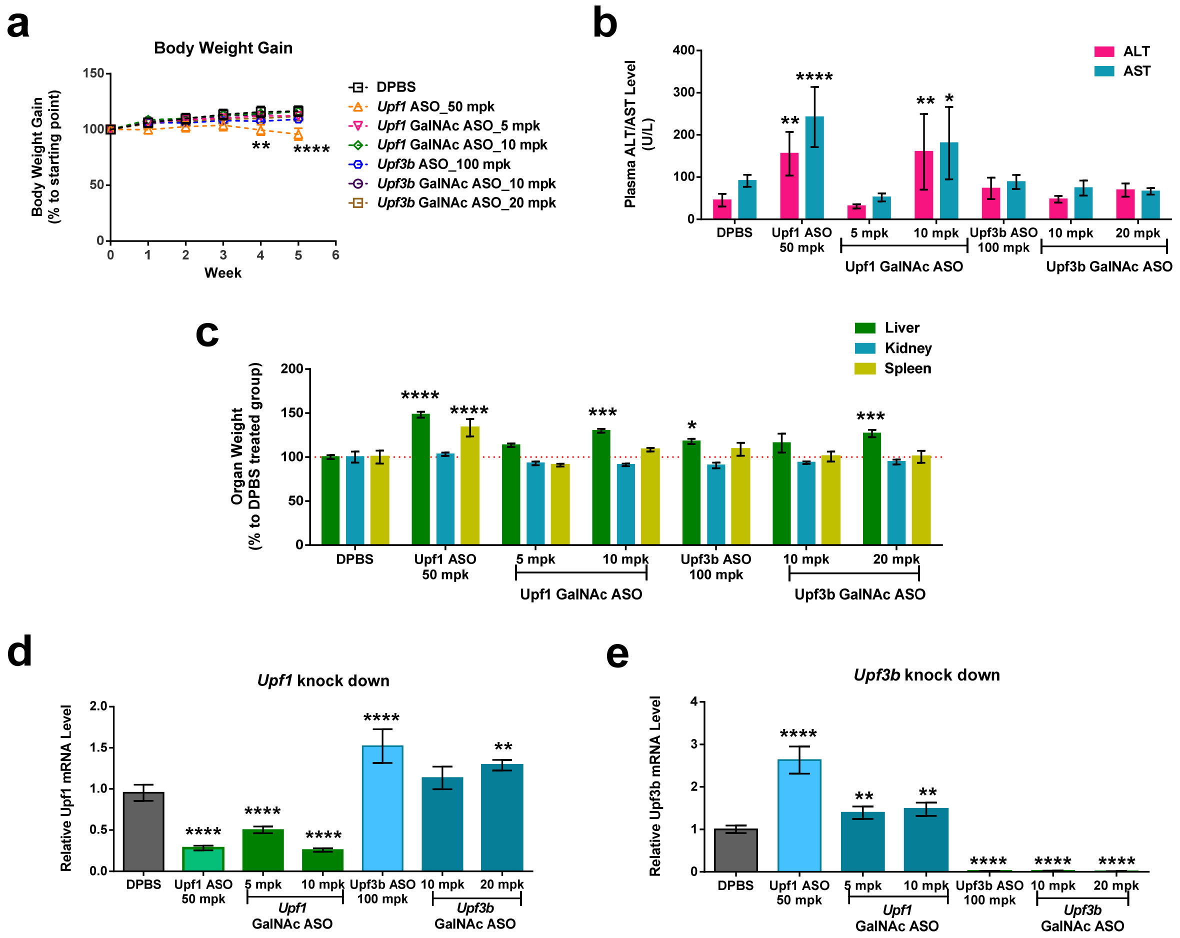
a
Body Weight Gain
Body Weight Gain (% to starting point)
Week
DPBS
Upf1 ASO_50 mpk
Upf1 GalNAc ASO_5 mpk
Upf1 GalNAc ASO_10 mpk
Upf3b ASO_100 mpk
Upf3b GalNAc ASO_10 mpk
Upf3b GalNAc ASO_20 mpk
b
Plasma ALT/AST Level (U/L)
ALT
AST
DPBS
Upf1 ASO 50 mpk
5 mpk
10 mpk
Upf1 GalNAc ASO
Upf3b ASO 100 mpk
10 mpk
20 mpk
Upf3b GalNAc ASO
c
Organ Weight (% to DPBS treated group)
Liver
Kidney
Spleen
d
Upf1 knock down
Relative Upf1 mRNA Level
e
Upf3b knock down
Relative Upf3b mRNA Level

Fig. S5

Figure S5. GalNAc-ASOs significantly improves ASO efficacy and tolerability in the liver.

Mice (n=4) were treated with DPBS, *Upf1*-ASO (50 mg/kg/week), *Upf1*-GalNAc-ASO (5 or 10 mg/kg/week), *Upf3b*-ASO (100 mg/kg/week), or *Upf3b*-GalNAc-ASO (10 or 20 mg/kg/week) every 5 days with 6 total doses for 4.5 weeks. Necropsy was performed 48 hrs after the last dose of ASO. Results are presented as means plus or minus standard errors. **a** Body weights measured once a week. **b** Plasma ALT and AST levels measured by clinical analyzer at necropsy. **c** Liver, kidney, and spleen weights measured at necropsy. **d, e** mRNA was purified from mouse liver and *Upf1* (**d**) or *Upf3b* (**e**) mRNA levels were measured by qPCR. *Gapdh* was used as an endogenous control. *Upf1* or *Upf3b* mRNA level in DPBS treated animals was set as 1. Statistical significance was determined using either a two-way ANOVA (panel **a**, **b**, and **c**) or a one-way ANOVA (panel **d** and **e**) and Dunnett's multiple comparison test in Prism. All groups were compared to DPBS-treated mouse group. *p<0.05; **p<0.01; ***p<0.001; ****p<0.0001.

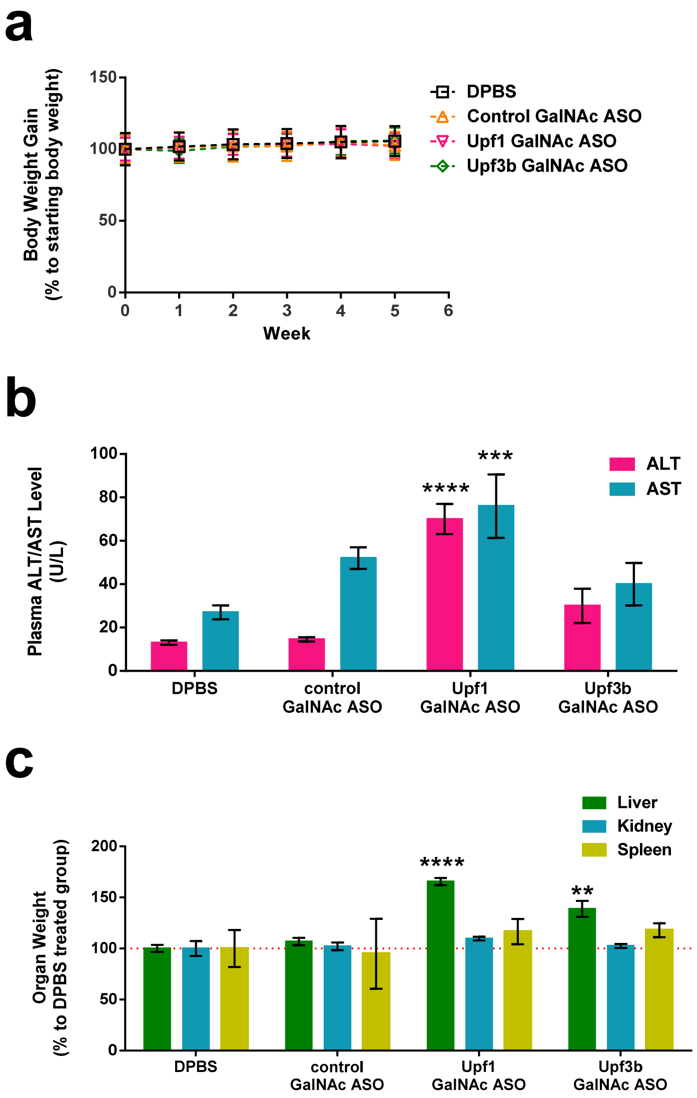
a
Body Weight Gain
(% to starting body weight)
150
100
50
0
0
1
2
3
4
5
6
Week
DPBS
Control GalNAc ASO
Upf1 GalNAc ASO
Upf3b GalNAc ASO
b
Plasma ALT/AST Level
(U/L)
100
80
60
40
20
0
****
***
ALT
AST
DPBS
control
GalNAc ASO
Upf1
GalNAc ASO
Upf3b
GalNAc ASO
c
Organ Weight
(% to DPBS treated group)
200
150
100
50
0
****
**
Liver
Kidney
Spleen
DPBS
control
GalNAc ASO
Upf1
GalNAc ASO
Upf3b
GalNAc ASO

Fig. S6

Figure S6. An *Upf3b*-GalNAc-ASO, but not an *Upf1*-GalNAc-ASO, is well tolerated in hemophilia mice. Study was performed as described in Figure 5. Results are presented as means plus or minus standard errors. **a** Body weights measured once a week. **b** Plasma ALT and AST levels measured by clinical analyzer at necropsy. **c** Liver, kidney, and spleen weights measured at necropsy. Statistical significance was determined using a one-way ANOVA and Dunnett's multiple comparison test in Prism. All groups were compared to DPBS-treated mice. ** $p<0.01$; *** $p<0.001$; ****$p<0.0001$.

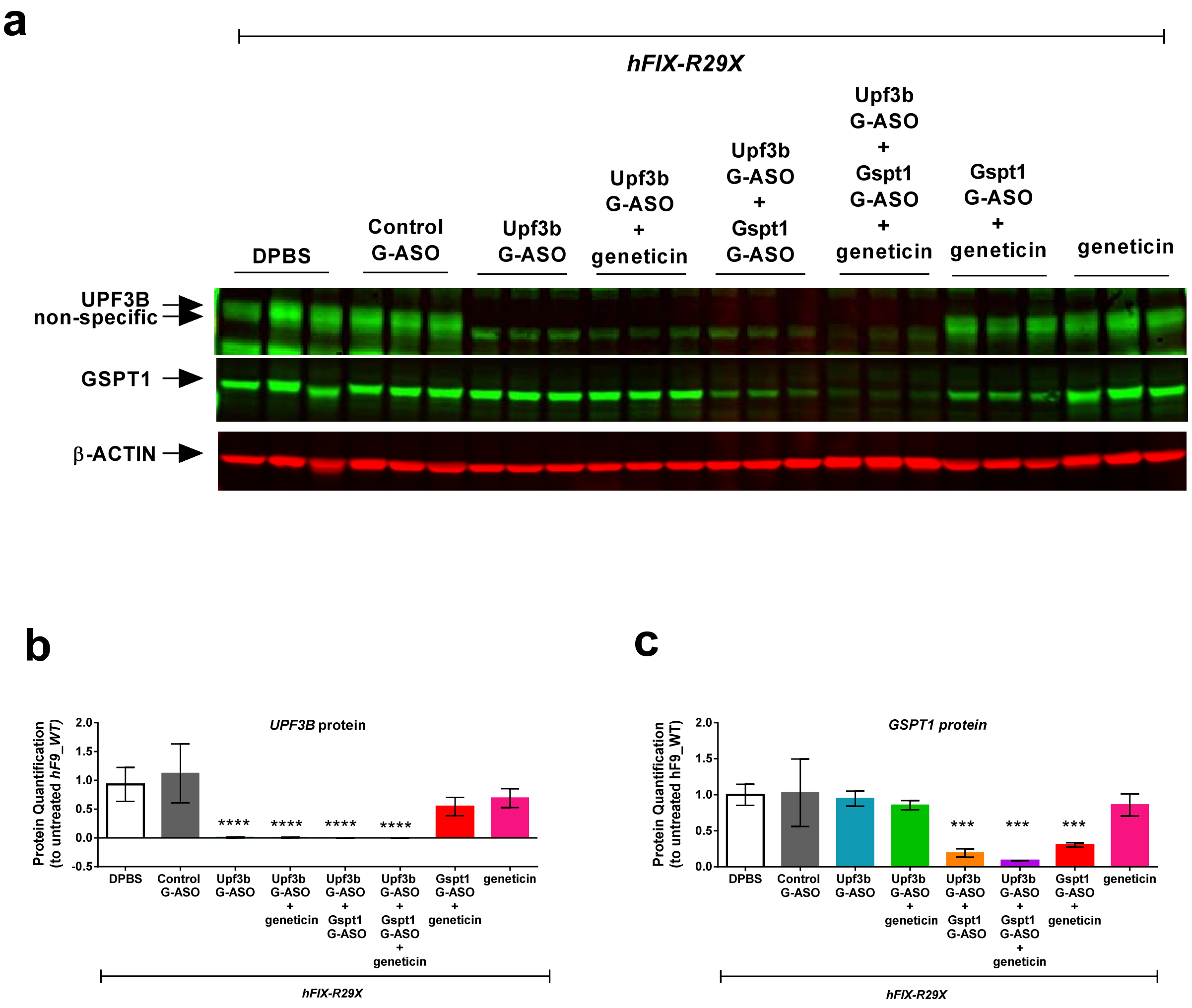
a
hFIX-R29X
DPBS
Control G-ASO
Upf3b G-ASO
Upf3b G-ASO + geneticin
Upf3b G-ASO + Gspt1 G-ASO
Upf3b G-ASO + Gspt1 G-ASO + geneticin
Gspt1 G-ASO + geneticin
geneticin
UPF3B
non-specific
GSPT1
β-ACTIN
b
UPF3B protein
Protein Quantification (to untreated hF9_WT)
2.0
1.5
1.0
0.5
0.0
-0.5
****
****
****
****
DPBS
Control G-ASO
Upf3b G-ASO
Upf3b G-ASO + geneticin
Upf3b G-ASO + Gspt1 G-ASO
Upf3b G-ASO + Gspt1 G-ASO + geneticin
Gspt1 G-ASO + geneticin
geneticin
hFIX-R29X
c
GSPT1 protein
Protein Quantification (to untreated hF9_WT)
2.0
1.5
1.0
0.5
0.0
***
***
***
DPBS
Control G-ASO
Upf3b G-ASO
Upf3b G-ASO + geneticin
Upf3b G-ASO + Gspt1 G-ASO
Upf3b G-ASO + Gspt1 G-ASO + geneticin
Gspt1 G-ASO + geneticin
geneticin
hFIX-R29X

Fig. S7

Figure S7. ASO-mediated depletion of UPF3B and GSPT1 in Hemophilia mice.

Study was performed as described in Figure 7. Western blot analysis was performed on mouse liver samples. **a** Odyssey images of the western blot. **b** and **c** Image studio quantification of the western blot image in **a**. β-ACTIN protein levels were used as a loading control. Results are presented as means plus or minus standard errors. Statistical significance was determined using a one-way ANOVA and Dunnett's multiple comparison test in Prism. All groups were compared to DPBS-treated mice. ***$p<0.001$; ****$p<0.0001$.
